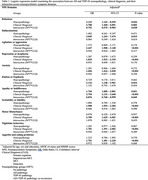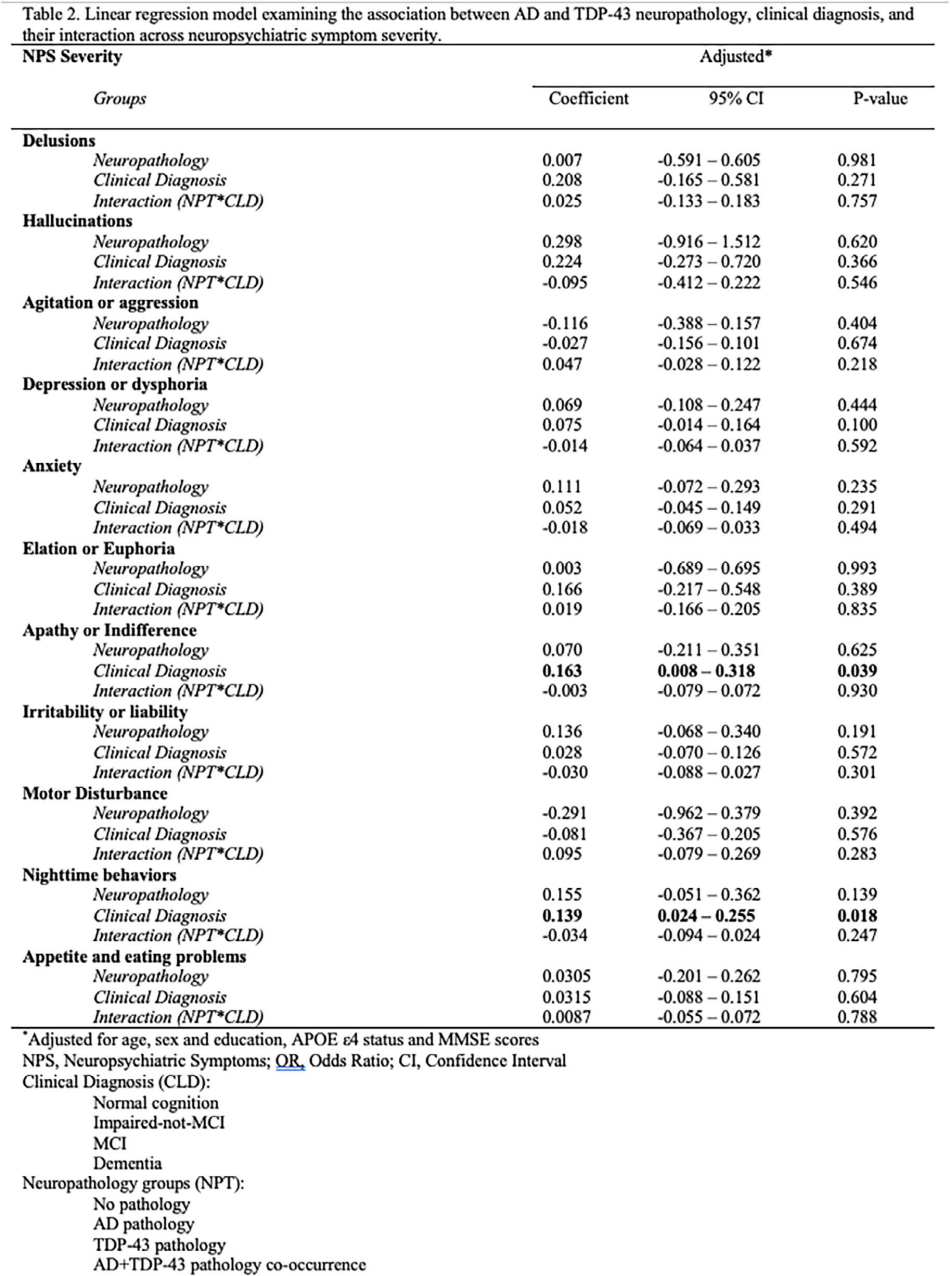# Examining the Interaction of Alzheimer's Disease and TDP‐43 Neuropathology, and Clinical Diagnosis on Neuropsychiatric Symptoms Burden

**DOI:** 10.1002/alz70857_103861

**Published:** 2025-12-25

**Authors:** Adrienne L Atayde, Neda Rashidi‐Ranjbar, Marc A Khoury, Francis A Fernandes, Luis R Fornazzari, Nathan W. Churchill, Simon J. Graham, Tom A. Schweizer, David G. Munoz, Corinne E. Fischer

**Affiliations:** ^1^ Keenan Research Centre for Biomedical Science, Li Ka Shing Knowledge Institute, St. Michael's Hospital, Toronto, ON, Canada; ^2^ Institute of Medical Science, Temerty Faculty of Medicine, University of Toronto, Toronto, ON, Canada; ^3^ Faculty of Medicine, Department of Neurology, University of Toronto, Toronto, ON, Canada; ^4^ Department of Medical Biophysics, University of Toronto, Toronto, ON, Canada; ^5^ Sunnybrook Research Institute, Toronto, ON, Canada; ^6^ Division of Neurosurgery, St. Michael's Hospital, Toronto, ON, Canada; ^7^ Division of Neurosurgery, Faculty of Medicine, University of Toronto, Toronto, ON, Canada; ^8^ Temerty Faculty of Medicine, Department of Laboratory Medicine and Pathobiology, University of Toronto, Toronto, ON, Canada; ^9^ Division of Pathology, St. Michael's Hospital, Toronto, ON, Canada; ^10^ Temerty Faculty of Medicine, Department of Psychiatry, University of Toronto, Toronto, ON, Canada

## Abstract

**Background:**

Neuropsychiatric symptoms (NPS) are prevalent in individuals with Alzheimer's disease (AD), impacting disease progression and patient quality of life. AD pathology (amyloid and tau) and related TDP‐43 neuropathology may contribute to NPS. The National Alzheimer's Coordinating Center (NACC) database was used to explore the relationship between NPS and AD, TDP‐43 neuropathology and clinical diagnosis.

**Method:**

A total of 1391 participants from the NACC database were included in the analysis, with data available on NPS (NPI‐Q domains), clinical diagnosis (normal cognition, impaired‐not‐MCI, MCI, and dementia) and neuropathology. Logistic and linear regression models assessed associations between NPS presence or absence, severity and interactions involving clinical diagnosis and AD and TDP‐43 neuropathology, including their co‐occurrence. Age, sex, education, cognitive scores, and APOE e4 status were adjusted for in all models. Odds ratios (ORs) and 95% confidence intervals (CIs) were computed to quantify the effects.

**Result:**

The regression models for several NPS domains were statistically significant with clinical diagnosis being the most consistent across domains (Table 1). The clinical diagnosis and neuropathology were significantly associated with apathy (OR = 2.80, 95% CI [2.13, 3.66], p < .001; OR = 1.74, 95% CI [1.09, 2.80], p = .021, respectively) and delusions (OR = 2.71, 95% CI [1.44, 5.09], p = .002; OR = 3.12, 95% CI [1.14, 8.54], p = .026, respectively). Interaction effects were also observed for apathy (OR = 0.88, 95% CI [0.77, 1.00], p = .049) and delusions (OR = 0.75, 95% CI [0.58, 0.98], p = .038). Clinical diagnosis also influenced NPS severity in nighttime behaviors (β = 1.14, 95% CI [0.02, 0.26], p = .018) and apathy (β = 0.16, 95% CI [0.01, 0.32], p = .039). Age, sex, education, cognitive scores, and APOE e4 status had domain‐specific effects.

**Conclusion:**

The findings highlight the role of clinical diagnosis on NPS across domains, with AD and TDP‐43 neuropathology and demographic factors exerting domain‐specific effects, most notably in delusions and apathy. Interaction effects between neuropathology and clinical diagnosis were only seen in apathy, suggesting that pathology and clinical diagnosis may have independent effects on NPS. Future research is warranted to examine these effects longitudinally.